# Age as a moderator in the interplay among locus of control, coping, and quality of life of people with chronic pain

**DOI:** 10.1093/pm/pnad079

**Published:** 2023-06-16

**Authors:** Joanna Kłosowska, Dominika Farley, Justyna Brączyk, Ewa Buglewicz-Przewoźnik, Przemysław Bąbel

**Affiliations:** Pain Research Group, Institute of Psychology, Jagiellonian University, 30-060 Kraków, Poland; Pain Research Group, Institute of Psychology, Jagiellonian University, 30-060 Kraków, Poland; Pain Research Group, Institute of Psychology, Jagiellonian University, 30-060 Kraków, Poland; Pain Research Group, Institute of Psychology, Jagiellonian University, 30-060 Kraków, Poland; Pain Research Group, Institute of Psychology, Jagiellonian University, 30-060 Kraków, Poland

**Keywords:** chronic pain, locus of control, coping strategies, age, moderated mediation

## Abstract

**Objective:**

Identifying the factors that determine the quality of life of patients with chronic pain is an integral part of developing interventions to reduce the negative impact of persistent pain. Locus of control (LoC) could play an important role in adaptation to prolonged pain, but the results of studies are inconsistent. We examined the link between pain LoC and quality of life. Moreover, we investigated whether the relationship between LoC and quality of life is mediated by passive and active coping, and whether age moderates the LoC–coping relationship.

**Methods:**

The study was cross-sectional, and variables (internal, chance and powerful-others LoC, pain coping strategies, average pain intensity, and quality of life) were assessed via questionnaires in a sample of 594 individuals (67% females) with chronic pain who were 18–72 (mean: 36) years of age.

**Results:**

Mediation and moderated mediation analyses were conducted. Internal and external LoC were associated, respectively, with better and with worse quality of life. Passive coping mediated the association between the powerful-others dimension of LoC and poor quality of life. Additionally, indirect effects of internal LoC on quality of life via passive and active coping were found. The relationship between the powerful-others dimension of LoC and coping was stronger for middle-aged and older individuals than for younger individuals.

**Conclusions:**

This study contributes to a better understanding of the mechanisms linking LoC with quality of life of patients with chronic pain. Depending on the age, control beliefs might translate differently into strategies used to cope with pain, and thus into quality of life.

## Introduction

Chronic pain conditions are among the leading causes of disability,^1^ affecting more than 30% of adults.^2^ A growing body of studies^3^^,^^4^ shows that chronic pain conditions pose a burden on the psychosocial functioning of individuals who suffer from them. Understanding which factors are associated with poor quality of life (QoL) is crucial for developing interventions directed at reducing the negative impact of persistent pain.^5^

The cognitive behavioral therapy model of chronic pain assumes that a person’s beliefs and expectations shape their reactions to pain, which in turn affects QoL.^5^^,^^6^ One type of belief that has been found to be related to the QoL of patients with chronic pain is *health locus of control* (LoC),^7^^,^^8^ which refers to a generalized expectancy about where control over health lies.^9^ Studies show that in patients with chronic pain, internal LoC (belief that our own behavior determines our health status) is associated with higher QoL.^5^^,^^8^ The relationship between external LoC (belief that health depends on external agents like other people or chance/fate/luck) and QoL is less clear, and the results of empirical investigations are inconsistent. Some studies suggest that patients with chronic pain who believe that their health is affected mainly by the actions of other people (the “powerful-others” dimension of LoC) have a level of QoL similar to that of patients with an internal LoC, and only the “chance” dimension of LoC has a detrimental effect on QoL.^8^ On the other hand, at least one study has indicated that a powerful-others LoC is not related to QoL,^10^ and another study^5^ showed that both dimensions of external LoC are related to QoL impairments among patients with chronic pain. In the light of these contradictory findings, more in-depth research on the LoC–QoL relationship is needed.

One possible mechanism that links dimensions of LoC with QoL could be the tendency to use specific methods of coping with pain. Previous research has suggested that patients who view health outcomes as being controlled by external factors (chance/fate or others) tend to rely on passive pain coping strategies (eg, catastrophizing) that are characterized by helplessness and relying on others, whereas those who believe they have control over their health use more active ways of coping.^11–13^ Active coping strategies (eg, diverting attention, behavioral activity) are often described as adaptive because they are related to greater well-being; passive strategies are seen as maladaptive because they have been shown to be linked to psychological distress.^14^

Nevertheless, the relationship between LoC and coping with pain is probably much more complex than most research on this topic suggests. For instance, a developmental component could be involved, with age acting as a potential moderator of the aforementioned association.^15^^,^^16^ A previous study^15^ has shown that, depending on participants’ age, LoC was differently related to coping strategies: Chance LoC was positively associated with distancing for older individuals but negatively for younger individuals, and internal LoC was negatively associated with escape–avoidance for older persons but positively for younger persons. When discussing their results, the authors of that study pointed out that the way older, and likely more mature, individuals deal with the situation might depend more strongly on their appraisal of controllability. In other words, if a stressful situation is perceived as something beyond their control, they rather choose a more passive way of dealing with it; however, if they perceive the situation as something controllable, they are more likely to engage in an active way of coping.

Congruence has been proposed as an important factor determining the effectiveness of coping (the so-called goodness-of-fit hypothesis).^17^ Literature suggests that, in general, active coping strategies like those focusing on solving the problem tend to work better with stressors considered to be controllable, whereas strategies focusing on emotions seem to be more effective for stressors seen as uncontrollable.^18^^,^^19^ As people age, they become more experienced in dealing with different problems. They encounter, assess, and respond to many different situations and stressors, and through this process they not only increase their coping repertoires but also can learn which strategies are generally ineffective and which work well in various scenarios.^20^ Therefore, we might expect that among older individuals, the relationship between the belief that they can control their pain and the use of active coping strategies and the relationship between the belief that mainly external factors influence their pain and the use of passive coping strategies would be more evident than among younger individuals. Unfortunately, studies examining the interplay between LoC, age, and coping strategies are almost nonexistent and are outdated.^15^^,^^16^ Further research is needed to shed more light on the role played by age in the LoC–coping association.

The present study aimed to: (1) examine the relationship between LoC and QoL among individuals suffering from chronic pain; (2) check whether active and passive coping mediates the LoC–QoL relationship; and (3) evaluate the moderating role of age in the relationship between LoC and active/passive coping.

The following hypotheses were formulated:

H1A. Internal LoC is associated with better QoL.

H1B. External LoC (powerful-others LoC, chance LoC) is associated with worse QoL.

H2A. Active coping is associated with better QoL.

H2B. Passive coping is related to worse QoL.

H3A. Internal LoC is associated positively with active coping and negatively with passive coping.

H3B. External LoC is associated positively with passive coping and negatively with active coping.

H4A. Active and passive coping strategies mediate the relationship between internal LoC and QoL.

H4B. Active and passive coping strategies mediate the relationship between external LoC and QoL.

**Figure 1.**
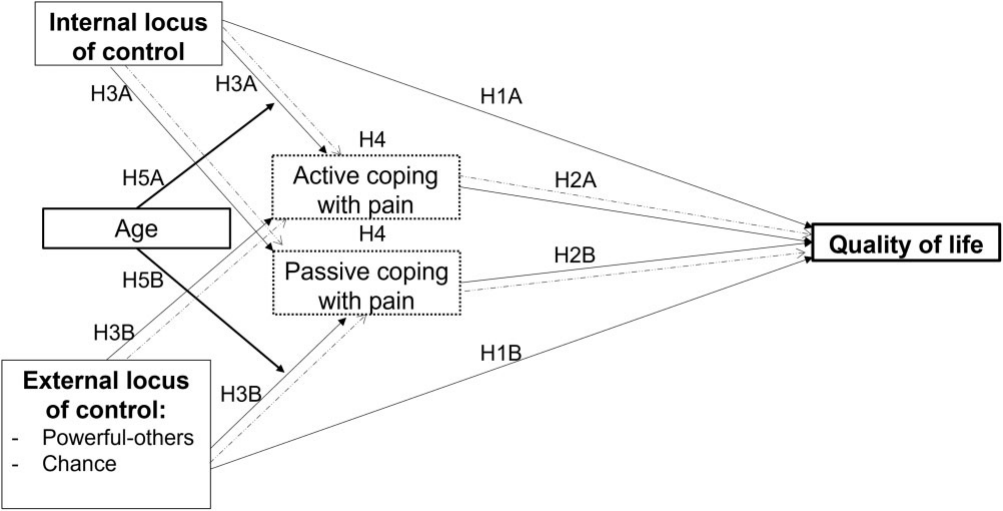
Conceptual model. The mediator variable and mediation effects are represented by dotted lines.

H5. Age moderates the relationship between internal/external LoC and coping with pain. In particular, because the way more mature individuals deal with stressors should be more congruent with their appraisal of controllability of the situation, the relationship between internal LoC and active coping (H5A), as well as between external LoC and passive coping (H5B), is stronger in older adults than in younger adults. See Figure 1 for details.

## Methods

### Participants

**Figure 2.**
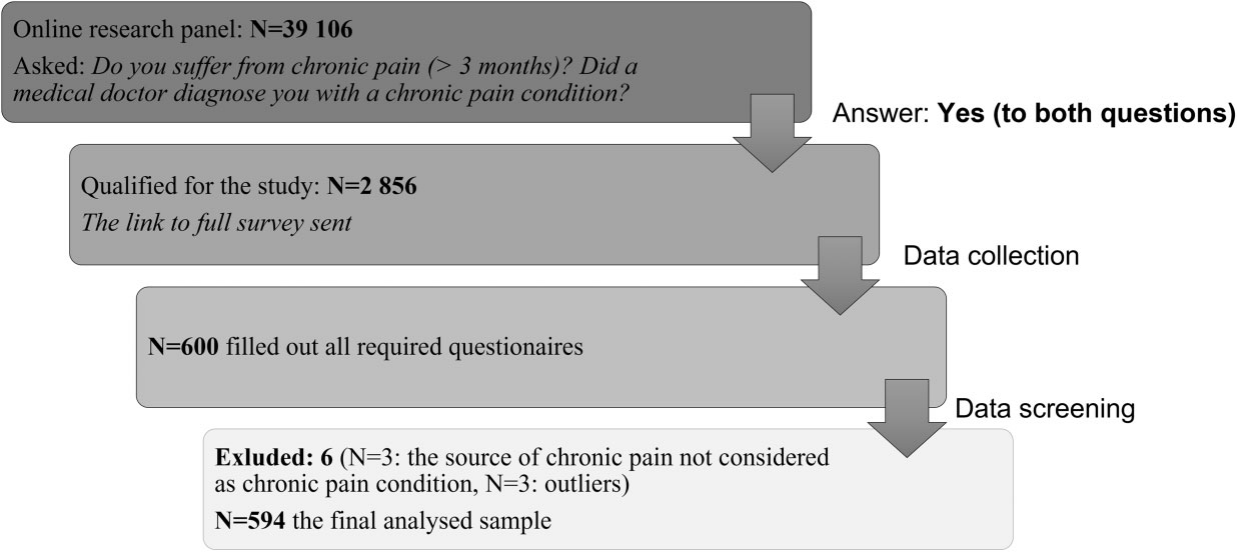
Sample recruitment flow chart.

A clinical research company contracted by the authors reached a total of 39 106 potential participants through an online research panel. The panel represented the Polish population of Internet users, and its demographic characteristics matched those of Polish Internet users found by the Polish Centre for Public Opinion Research,^21^ a governmental opinion polling institute. Panelists who declared that they suffered from a chronic pain condition (ie, had experienced pain for more than 3 months) and who answered “yes” to the question “Did a medical doctor diagnose you with a chronic pain condition?” were preliminarily qualified for the study (*n* = 2856; 7.3%). The final sample was narrowed down to the panelists who answered all the items (*n* = 600; no missing data). Six participants were excluded from the study (2 males, 4 females) because the medical condition they declared as the source of their chronic pain was not considered to be a chronic pain condition (*n* = 3: schizophrenia, thyroid condition, and premenstrual syndrome) (see Figure 2) or they were identified as outliers (*n* = 3).

The remaining sample of 594 participants consisted of 33% males and 67% females. This ratio is consistent with other studies on chronic pain.^22–24^ The average age of participants was 35.75 (SD = 11.63) years. Age distribution can be found in the Supplementary Material. The majority of participants were professionally active or studying (85.7%); the remaining participants were retired (4.2%), unemployed (5.7%), or received health-related benefits (4.4%) (see Table 1).

**Table 1.** Sociodemographic, pain-related, and healthcare characteristics of the sample.

	Full sample	Women	Men
	(*n* = 594)	(*n* = 398)	(*n* = 196)
Demographic characteristics			
Age, years, mean (SD)	35.75 (11.63)	34.73 (11.09)	37.81 (12.37)
Employment status, % (*n*)			
Professionally active or studying	85.7 (509)	85.9 (342)	85.2 (167)
Retired	4.2 (25)	3.3 (13)	6.1 (12)
Pensioners	4.4 (26)	3.3 (13)	6.6 (13)
Unemployed	5.7 (34)	7.5 (30)	2 (4)
Professional activity, % (*n*)			
White-collar workers	52.5 (312)	54.3 (216)	49 (96)
Physical activity	31.8 (189)	29.4 (117)	36.7 (72)
No professional activity	15.7 (93)	16.3 (65)	14.3 (28)
Pain location, % (*n*)			
Back pain	60.3 (358)	58.8 (234)	63.3 (124)
Headaches	35.5 (211)	39.9 (159)	26.5 (52)
Joints	26.3 (156)	25.6 (102)	27.6 (54)
Muscles	17.8 (106)	16.8 (67)	19.9 (39)
Internal organs	9.1 (54)	9.8 (39)	7.7 (15)
Jaw	3 (18)	2 (8)	5.1 (10)
Pain intensity during the prior week, mean (SD)			
Average	5.72 (2)	5.7 (2.01)	5.75 (1.96)
Maximum	7 (1.88)	7.13 (1.86)	6.69 (1.88)
Healthcare provider, % (*n*)			
Medical doctor only	35 (208)	36.4 (145)	32.14 (63)
Other specialist only	14.3 (85)	11.8 (47)	19.4 (38)
Medical doctor and other specialist	50.7 (301)	51.8 (206)	48.5 (95)

Column values indicate % (*n*) for categorical variables or mean (SD) for continuous variables. Average and maximum pain intensity was measured on a 0-to-10 numeric rating scale.

### Procedure

The participants were asked to fill in the online questionnaires (listed in the “Measures” section) at a time they found convenient. Data were collected across 2 months (May–June 2019). The participants who completed the study were compensated financially (they received points from a medical research company, which they could exchange for money later). All participants expressed their informed consent in a digital form. The study protocol was reviewed and approved by the Ethics Committee of the Institute of Psychology at the Jagiellonian University.

### Measures

The online survey consisted of 4 sets of questions: (1) demographic data (sex, age, employment status, and professional activity); (2) questions related to pain location, duration, and intensity; (3) information about medical care; and (4) questions about seeking psychological help to treat chronic pain. Moreover, validity check questions were inserted into a survey to filter out respondents who were not answering honestly or carefully. Because of the limited scope of the present article, the analysis of data related to treatment is described in a separate publication.^25^

Within the survey, Polish adaptations of 3 questionnaires were applied:

- *Coping Strategies Questionnaire.* The Coping Strategies Questionnaire was developed to measure the use of coping strategies by patients suffering from pain.^26^ It consists of 42 statements describing ways of dealing with pain and 2 questions assessing one’s own ability to cope with and reduce pain. In the Polish version, the statements are combined into 6 subscales reflecting 6 strategies of dealing with pain.^21^ Each of the strategies can also be categorized as active or passive.^13^^,^^14^ Active coping (such as: diverting attention, eg, “I try to think of something pleasant”; reinterpretation of pain, eg, “I imagine that the pain is outside of my body”; ignoring pain, eg, “I just go on as if nothing happened”; coping self-statements, eg, “I tell myself that I can overcome the pain”; behavioral activity, eg, “I try to be around other people”) relates to a patient’s proactive attempts to control or to function despite their pain. Passive coping (such as: catastrophizing, eg, “It is terrible and I feel it’s never going to get any better”; praying/hoping, eg, “I pray for the pain to stop”) relates to helplessness, giving up control over pain, and letting pain become an immanent part of life. Active and passive coping scores were calculated with the system developed by Nicholas et al.^13^ and successfully applied by Snow-Turek, Norris, and Tan.^14^ The score falls between 0 and 180 points for active coping and between 0 and 72 points for passive coping. The Polish adaptation of the questionnaire has been validated.^27^
- *Beliefs about Pain Control Questionnaire.* The Beliefs about Pain Control Questionnaire is a 13-item tool measuring beliefs about controlling pain. It assesses the strength of individual beliefs about controlling pain and comprises 3 subscales measuring the power of personal beliefs about factors influencing one’s pain: personal control (internal LoC), eg, “I am directly responsible for my pain” (participants can obtain from 5 to 30 points in this subscale); powerful-others LoC, eg, “Whether or not I am in pain depends on what the doctors do for me” (from 4 to 24 points); and chance LoC, eg, “Being pain-free is largely a matter of luck” (from 4 to 24 points).^28^ The Polish adaptation of the questionnaire has been validated.^27^
- *Short Form-36 Health Survey.* The Short Form-36 Health Survey measures health-related QoL. It contains 36 statements, which form 8 subscales that assess the following: physical functioning (“Does your health now limit you in these activities? If so, how much?”), role limitations due to physical health problems (“During the past 4 weeks, have you had any of the following problems with your work or other regular daily activities as a result of your physical health?”), bodily pain (eg, “How much bodily pain have you had during the past 4 weeks?”), general health perceptions (eg, “In general, would you say your health is …”), vitality (eg, “Did you have a lot of energy?”), social functioning (eg, “During the past 4 weeks, how much of the time have your physical health or emotional problems interfered with your social activities?”), role limitations due to emotional problems (“During the past 4 weeks, have you had any of the following problems with your work or other regular daily activities as a result of any emotional problems?”), and general mental health (eg, “Have you felt so down in the dumps that nothing could cheer you up?”).^29^ The Polish version of the questionnaire was used in the present study.^30^ It allows the assessment of QoL on one combined scale, with scores falling between 0 and 171 points. The highest point value indicates the lowest level of QoL.
  Cronbach’s alpha and McDonald’s omega reliability coefficients for each scale, obtained in the present sample, can be found in Table 2.
- *Pain intensity measure.* The average pain intensity and maximum pain intensity within the prior week were measured on a numeric rating scale, ranging from 0 (“no pain at all”) to 10 (“the most pain that I have ever experienced”). For the purposes of our study, we chose to include the average pain intensity rating for the previous week as a more representative index.^31^^,^^32^

**Table 2.** Reliability statistics for the questionnaires used in the study calculated for the studied sample.

Scale	McDonald’s omega	Cronbach’s alpha
BPCQ—internal control	0.76	0.75
BPCQ—powerful others	0.79	0.79
BPCQ—chance/fate	0.59	0.58
SF-36	0.89	0.85
CSQ—diverting attention	0.82	0.81
CSQ—reinterpretation of pain	0.89	0.88
CSQ—ignoring pain	0.88	0.88
CSQ—coping self-statements	0.84	0.84
CSQ—behavioral activity	0.84	0.84
CSQ—catastrophizing	0.87	0.87
CSQ—praying/hoping	0.82	0.82
CSQ—active coping	0.96	0.96
CSQ—passive coping	0.88	0.88

*n* = 594.

Abbreviations: BPCQ = Beliefs about Pain Control Questionnaire; CSQ = Coping Strategies Questionnaire; SF-36 = Short Form-36 Health Survey.

### Statistical analyses

Analyses were carried out in IBM SPSS Statistics for Windows, Version 28.0. (IBM Corp., 2021, Armonk, NY, United States). Before the primary analyses were conducted, an assessment of common method bias was performed. Second, the bivariate associations among the study variables were examined with Pearson correlation analyses. Next, mediation analysis was conducted with the PROCESS macro v.4.1.^33^ Because this macro does not allow the assessment of indirect effects for 3 independent variables at once, 3 parallel analyses (model 4) were conducted, as recommended by Hayes,^33^ to overcome this limitation. In each analysis, one dimension of LoC was introduced as the independent variable (*X*); active coping and passive coping were mediator variables (*M*); QoL was the dependent variable (*Y*); and the rest of the LoC dimensions were included as covariates. In this way, all parameters of the model, with 3 independent variables and 2 mediators, could be assessed. Also, the variables that have been shown in previous studies to be related to coping and QoL (sex, average pain intensity in the previous week) were controlled for in these analyses. To establish that mediation had occurred, the analytical procedure suggested by MacKinnon and Luecken^34^ was applied, requiring (1) a significant relationship between independent and dependent variables; (2) a significant association between the mediator and the dependent variable while controlling for the independent variable; and (3) a significant coefficient for the indirect path between independent variable and the dependent variable via the mediator. The indirect effects were tested with bias-corrected bootstrapping (*n* = 5000) and 95% confidence intervals.

In the moderated mediation analyses (model 7 in PROCESS), the previous variables were kept in the models, but age was added as the moderator (*W*). Predictors were mean-centered before creation of the interaction term. To probe significant interaction effects, simple-slopes analysis (pick-a-point approach) was performed: The conditional effect of LoC on coping was tested at low (mean – 1 SD), medium (mean) and high (mean + 1 SD) levels of age.

## Results

### Assessment of common method bias

Harman’s single-factor test^35^ was used to identify common method variance. All the items of the questionnaires used in the study were introduced into exploratory factor analysis (maximum-likelihood method), and the unrotated solution was examined. The Kaiser–Meyer–Olkin value was 0.91, which shows that the sample was very well suited for conducting factor analysis. The principal factor explained 17.70% of variance, which suggests that there was no serious common method bias problem in the study.

### Pain-related background data

The majority of participants suffered from some form of back pain (60.3%); 35.5% suffered from headaches, 26.3% indicated joints as the source of chronic pain, and 17.8% indicated muscles as the source of chronic pain. Only 9.1% declared internal organs as the source of pain, and 3% declared the jaw. Half of all participants (49.2%) suffered from more than one chronic pain condition. The average pain intensity reported by the participants in the week preceding the study was rated as 5.72 (SD = 2), and the maximum pain was 7 (Table 1).

### Correlations and descriptive statistics

Internal LoC was associated with better QoL, and both dimensions of external LoC were related to worse QoL. Passive (*r* = 0.24, *P* < .001) but not active coping showed a significant association with worse QoL. Internal LoC correlated positively with active (*r* = 0.32, *P* < .001) and passive coping (*r* = 0.22. *P* < .001). A similar pattern of relationships was observed for both dimensions of external LoC: They correlated positively with passive (chance LoC: *r* = 0.44, *P* < .001; powerful-others LoC: *r* = 0.40, *P* < .001) and active coping (chance LoC: *r* = 0.29, *P* < .001; powerful-others LoC: *r* = 0.25, *P* < .001). Age correlated significantly, although very weakly, with worse QoL (*r* = 0.13, *P* < .01), lower internal LoC (*r* = –0.09, *P* < .05), and higher powerful-others LoC (*r* = 0.16, *P* < .001). Descriptive statistics and complete results of the correlation analyses are presented in Table 3.

**Table 3.** Descriptive statistics and results of correlational analysis (Pearson’s *r*).

	Min/Max	S (SD)	K (SD)	M (SD)	(1)	(2)	(3)	(4)	(5)	(6)	(7)	(8)	(9)
BPCQ chance **(1)**	4/24	–0.25 (0.10)	0.15 (0.20)	14.66 (3.78)	1	0.43***	0.24***	0.29***	0.44***	0.19***	0.10*	–0.04	–0.02
BPCQ powerful others **(2)**	4/24	–0.09 (0.10)	0.01 (0.20)	14.21 (4.12)		1	0.20***	0.25***	0.40***	0.24***	0.16***	0.16***	0.08*
BPCQ internal **(3)**	5/30	–0.17 (0.10)	0.13 (0.20)	17.92 (4.71)			1	0.32***	0.22***	0.02	–0.12**	–0.09*	0.13***
CSQ active (**4)**	0/174	–0.23 (0.10)	0.13 (0.20)	89.67 (32.88)				1	0.47***	0.16***	–0.03	0.05	0.10*
CSQ passive **(5)**	0/72	–0.14 (0.10)	–0.13 (0.20)	37.47 (13.73)					1	0.25***	0.24***	0.02	0.01
Pain intensity **(6)**	0/10	–0.04 (0.10)	–0.28 (0.20)	5.72 (2.00)						1	0.31***	0.14***	0.01
Low QoL **(7)**	11/159	–0.02 (0.10)	–0.42 (0.20)	85.41 (25.93)							1	0.13**	–0.11**
Age **(8)**	18/72	0.77 (0.10)	–0.02 (0.20)	35.75 (11.63)								1	0.13**
Sex **(9)**	—	—	—	—									1

*n* = 594;

* *P* < .05;

** *P* < .01;

*** *P* < .001.

Women were coded as 0; men were coded as 1.

Abbreviations: BPCQ = Beliefs about Pain Control Questionnaire; BPCQ chance = chance dimension of LoC measured by the BPCQ; BPCQ powerful others = powerful-others dimension of LoC measured by the BPCQ; BPCQ internal = internal LoC measured by the BPCQ; CSQ = Coping Strategies Questionnaire; CSQ active = active coping strategies measured by the CSQ; CSQ passive = passive coping strategies measured by the CSQ; K = kurtosis; LoQ = locus of control; low QoL = low quality of life measured by the Short Form-36 Health Survey; pain = average pain intensity in the previous week; QoL = quality of life; S = skewness.

### Mediation analyses

#### Indirect effects of internal LoC on QoL

**Figure 3.**
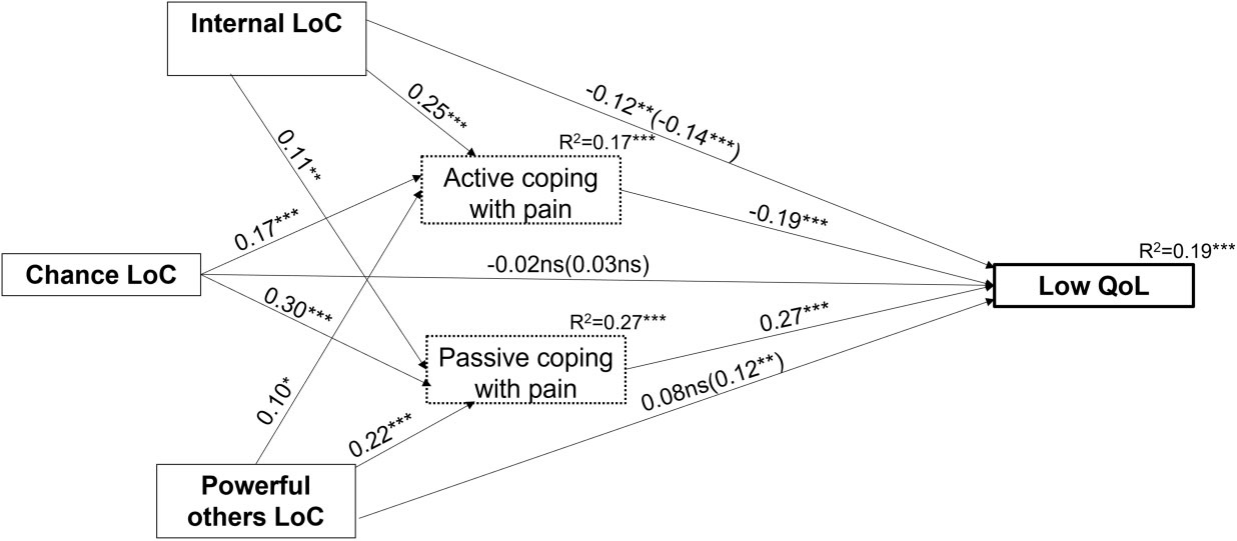
Results of mediation analyses in the form of a statistical diagram. *n* = 594, *P < .05, **P < .01, ***P < .001. Presented statistics are estimates calculated with the PROCESS macro and ordinary least squares regression-based approach as described in Hayes.^26^ Standardized coefficients are presented. The effects on the direct path from locus of control (LoC) to low quality of life (QoL) depict the direct effect and the total effect, respectively. Sex and average pain intensity in the prior week were controlled for in the analysis. The standardized indirect effect of internal LoC via active coping: β = –0.05, SE = 0.01, 95% CI: –0.07 to –0.02. The standardized indirect effect of internal LoC via passive coping: β = 0.03, SE = 0.01, 95% CI: 0.01 to 0.05. The standardized indirect effect of powerful-others LoC via active coping: β = –0.02, SE = 0.01, 95% CI: –0.04 to 0.001. The standardized indirect effect of powerful-others LoC via passive coping: β = 0.06, SE = 0.02, 95% CI: 0.03 to 0.09. The indirect effect of chance LoC via passive and active coping on QoL was not analyzed because the total effect was not significant.

The overall mediation model, which predicts QoL from internal LoC and active and passive coping, was significant and explained 19% of QoL variance (F [7586] = 19.14; *P* < .001). The direct effect of internal LoC on QoL was negative and significant (*P* < .01), which indicates that higher internal LoC is associated with better QoL. Internal LoC was positively associated with passive coping (*P* < .001), which was related to worse QoL (*P* < .001). The indirect effect of internal LoC on QoL via passive coping was significant (*b* = 0.16, SE = 0.07, 95% CI = 0.03 to 0.31), thus indicating partial mediation. Additionally, internal LoC was positively associated with active coping (*P* < .001), which in turn was related to better QoL (*P* < .001). The indirect effect of internal LoC on QoL through active coping was significant (*b* = –0.25, SE = 0.08, 95% CI = –0.41 to –0.12), thus suggesting partial mediation (see Figure 3).

#### Indirect effects of the powerful-others dimension of external LoC on QoL

The total effect of the powerful-others LoC on poor QoL was significant and positive (*P* < .01); however, after the mediators were included in the model, the effect proved to be insignificant (*P* = .09). Powerful-others LoC correlated positively with passive coping (*P* < .001), which was associated with worse QoL. The indirect effect of powerful-others LoC on QoL via passive coping was significant (*b* = 0.37, SE = 0.10, 95% CI = 0.20 to 0.59), thus indicating full mediation. Although powerful-others LoC was positively associated with active coping (*P* < .05), which was significantly related to better QoL, the indirect effect of powerful-others LoC on QoL through active coping was insignificant (*b* = –0.11, SE = 0.07, 95% CI = –0.28 to 0.01).

#### Indirect effects of the chance dimension of external LoC on QoL

The total effect of the chance dimension of LoC on QoL was insignificant (*b* = 0.17, SE = 0.30, 95% CI = –0.41 to 0.76; *P* = .56). As the first condition of MacKinnon & Luecken’s^34^ procedure to establish mediation was not met, the indirect effects were not analyzed further.

### Moderated mediation analyses

The interaction effects of internal LoC and age on active and passive coping were insignificant. Similarly, age did not moderate the relationship between chance LoC and both types of coping or the relationship between powerful-others LoC and active coping (all *P* > .49). However, there was a significant interaction effect of powerful-others LoC and age on passive coping (*b* = 0.02, SE = 0.01, 95% CI = 0.004 to 0.04; *P* < .05). The results of the analyses can be found in Table 4.

**Table 4.** Results of moderation analyses.

Independent variable	Dependent variable	Predictors included in the model	Estimate	SE	Bootstrap (*n* =5000)		*P*	*R^2^ (*Δ*R^2)^*
					95% CI _low_	95% CI _high_		
Chance LoC	Active coping	Intercept	37.62	7.28	23.33	51.91	<.001	0.17*** (0.00)
		Chance LoC	1.49	0.37	0.76	2.23	<.001	
		Age	0.07	0.11	–0.15	0.28	.55	
		Chance LoC×age	–0.00	0.03	–0.06	0.06	.95	
		*Average pain*	*1.59*	*0.65*	*0.33*	*2.86*	*<.05*	
		*Sex*	*4.03*	*2.68*	–*1.24*	*9.30*	*.13*	
		*Powerful-others LoC*	*0.75*	*0.35*	*0.07*	*1.43*	*<.05*	
		*Internal LoC*	*1.73*	*0.28*	*1.18*	*2.27*	*<.001*	
	Passive coping	Intercept	16.36	2.85	10.77	21.94	<.001	0.27*** (0.00)
		Chance LoC	1.07	0.15	0.79	1.36	<.001	
		Age	–0.02	0.04	–0.10	0.07	.70	
		Chance LoC×age	–0.01	0.01	–0.03	0.01	.50	
		*Average pain*	*0.94*	*0.25*	*0.45*	*1.44*	*<.001*	
		*Sex*	–*0.49*	*1.05*	–*2.55*	*1.57*	*.64*	
		*Powerful-others LoC*	*0.74*	*0.14*	*0.47*	*1.00*	*<.001*	
		*Internal LoC*	*0.30*	*0.11*	*0.09*	*0.51*	*<.01*	
Powerful-others LoC	Active coping	Intercept	25.89	7.56	11.06	40.73	<.001	0.17*** (0.00)
		Powerful-others LoC	0.74	0.35	0.06	1.42	<.05	
		Age	0.06	0.11	–0.16	0.28	.58	
		Powerful-others LoC×age	0.02	0.03	–0.03	0.07	.49	
		*Average pain*	*1.57*	*0.64*	*0.31*	*2.84*	*<.05*	
		*Sex*	*4.16*	*2.69*	–*1.13*	*9.44*	*.12*	
		*Chance LoC*	*1.49*	*0.37*	*0.76*	*2.22*	*<.001*	
		*Internal LoC*	*1.76*	*0.28*	*1.21*	*2.30*	*<.001*	
	Passive coping	Intercept	10.55	2.94	4.77	16.33	<.001	0.28*** (0.01*)
		Powerful-others LoC	0.74	0.13	0.47	1.00	<.001	
		Age	–0.02	0.04	–0.11	0.06	.59	
		Powerful-others LoC×age	0.02	0.01	0.00	0.04	.02	
		*Average pain*	*0.91*	*0.25*	*0.42*	*1.40*	*<.001*	
		*Sex*	–*0.32*	*1.05*	–*2.37*	*1.74*	*.76*	
		*Chance LoC*	*1.07*	*0.15*	*0.78*	*1.35*	*<.001*	
		*Internal LoC*	*0.33*	*0.11*	*0.12*	*0.55*	*<.01*	
Internal LoC	Active coping	Intercept	46.64	6.27	34.33	58.95	<.001	0.17*** (0.00)
		Internal LoC	1.72	0.28	1.17	2.27	<.001	
		Age	0.07	0.11	–0.15	0.29	.53	
		Internal LoC×age	0.01	0.02	–0.04	0.05	.72	
		*Average pain*	*1.60*	*0.64*	*0.33*	*2.86*	*<.05*	
		*Sex*	*4.01*	*2.69*	–*1.26*	*9.28*	*.14*	
		*Chance LoC*	*1.49*	*0.37*	*0.76*	*2.22*	*<.001*	
		*Powerful-others LoC*	*0.76*	*0.35*	*0.08*	*1.45*	*<.05*	
	Passive coping	Intercept	6.14	2.45	1.33	10.96	<.05	0.27*** (0.00)
		Internal LoC	0.31	0.11	0.09	0.52	<.01	
		Age	–0.02	0.04	–0.11	0.07	.66	
		Internal LoC×age	–0.00	0.01	–0.02	0.02	.81	
		*Average pain*	*0.94*	*0.25*	*0.44*	*1.43*	*<.001*	
		*Sex*	–*0.48*	*1.05*	–*2.54*	*1.58*	*.65*	
		*Chance LoC*	*1.07*	*0.15*	*0.78*	*1.35*	*<.001*	
		*Powerful-others LoC*	*0.74*	*0.14*	*0.47*	*1.01*	*<.001*	

*n*=594.

Controlled variables are presented in italics.

Abbreviations: LoC = locus of control; QoL = quality of life.

**Figure 4.**
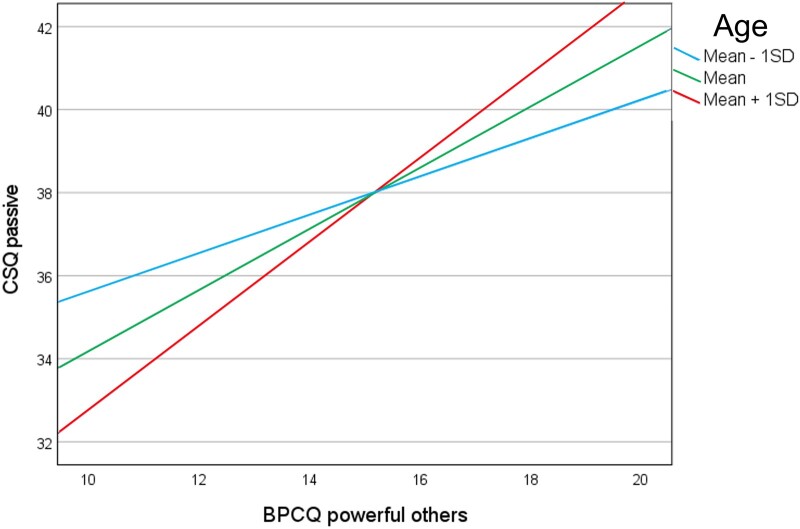
Simple-slopes analysis of age as a moderator in the association between the powerful-others dimension of locus of control (LoC) and passive coping. The relationship between powerful-others LoC and passive coping was stronger for middle- and older-aged participants (*b* = 1.01, SE = 0.18, 95% CI: 0.67 to 1.36; *P* < .001) than for those with average age (mean: *b* = 0.74, SE = 0.14, 95% CI: 0.47 to 1.00; *P* < .001) and young age (mean – 1 SD: *b* = 0.46, SE = 0.18, 95% CI: 0.11 to 0.82; *P* < .05). The index of moderated mediation was also significant (index = 0.01, SE = 0.01, 95% CI: 0.002 to 0.03). The indirect effect of powerful-others LoC through passive coping was stronger for middle- and older-aged individuals than for younger individuals (mean age + 1 SD: *b* = 0.51, SD = 0.14, 95% CI: 0.28 to 0.81; mean age: *b* = 0.38, SD = 0.10, 95% CI: 0.20 to 0.60; mean age – 1 SD: *b* = 0.23, SD = 0.11, 95% CI: 0.05 to 0.48).

The simple-slope analysis (Figure 4) indicated that the relationship between powerful-others LoC and passive coping was stronger for participants who were more than 47 years of age (1 SD above sample mean) than for those with average (ie, sample mean = 36 years) and young (1 SD below the mean = 24 years) age. The index of moderated mediation was also significant (index = 0.01, SE = 0.01, 95% CI = 0.002 to 0.03). The indirect effect of powerful-others LoC on QoL through passive coping was conditional on age and was stronger for middle- and older-aged individuals (*b* = 0.51, SD = 0.14, 95% CI = 0.28 to 0.81) than for younger individuals (mean age – 1 SD: *b* = 0.23, SD = 0.11, 95% CI = 0.05 to 0.48).

## Discussion

In the present study, we examined the effect of LoC on the QoL of patients with chronic pain, as well as the roles of coping and age in the association between LoC and QoL. Our findings indicated that internal LoC and active forms of coping with pain were positively related with QoL, whereas external LoC and passive coping were negatively related with QoL. Moreover, the relationship between the powerful-others dimension of LoC and QoL was mediated by passive coping, and the association between internal LoC and QoL was mediated by both active and passive coping. Interestingly, the positive association between powerful-others LoC and passive coping, as well as an indirect effect of powerful-others LoC on QoL via passive coping, were more potent for individuals who were more than 47 years of age than for younger individuals.

In line with our hypothesis (H1A), we found that internal LoC was associated with better QoL among people with chronic pain. This result is not surprising, given that individuals who believe they can influence their pain have higher motivation to engage in various health behaviors that improve QoL.^36^ It was also assumed (H1B) that a stronger belief that pain is controlled by external factors (chance LoC) is associated with worse QoL. This hypothesis was confirmed when the bivariate relationship between the variables was analyzed; however, it turned out to be insignificant when the other variables were controlled for in the regression analyses, thus suggesting that chance LoC did not explain QoL over and above other predictors. Although the results of previous studies showed that chance LoC is associated with poorer well-being and more severe depression in patients with chronic pain,^8^^,^^10^ it is worth mentioning that LoC dimensions were analyzed independently from one another in those studies. In the future, the chance LoC–QoL link needs to be more closely studied with multivariate analytic techniques. In the present study, powerful-others LoC was also related to worse QoL: It has been suggested previously that individuals who think that their pain is controlled by physicians might rely too much on their doctors, but this comes at the expense of self-management behaviors that are necessary for the successful treatment of conditions associated with persistent pain.^5^

The second hypothesis was also confirmed: Active coping was related to better (H2A) QoL, and passive coping was related to worse (H2B) QoL among patients with chronic pain. These findings are consistent with previous research indicating that active coping is linked to better adaptation, and passive coping is associated with psychological distress and increased pain.^14^^,^^37^

The third hypothesis was only partially supported: As predicted, internal LoC correlated positively with active coping (H3A), and both dimensions of external LoC correlated positively with passive coping strategies (H3B). However, contrary to our expectations, the relationships between internal LoC and passive coping and between external LoC and active coping were also positive, but weaker. These results could reflect dynamic and bidirectional relationships between LoC and coping. Although people with internal LoC might be more likely to choose active coping, if their efforts result in failure, this can strengthen their belief that their condition depends on external factors. Similarly, when passive coping strategies are effective, people could start to believe that they can influence their pain, thus producing a positive correlation between internal LoC and passive coping.^12^ Also, other variables unaccounted for in the present study could explain these associations, with religiosity being one of them. Previous studies have shown that internal LoC is positively related to intrinsic religiosity,^38^ as well as to religious behaviors like asking for help from a divine power.^39^ It is possible that for religious individuals, praying is viewed as an active effort that implies recognition of divine power without a relinquishment of personal autonomy or loss of internal control.^38^^,^^40^ As this study was cross-sectional and did not control for the level of religiosity, these effects cannot be untangled. Longitudinal studies accounting for potential mediator variables are needed to fully understand these links.

Passive coping fully mediated the relationship between powerful-others LoC and QoL (H4). This is consistent with the interpretation^41^ that people with external LoC do not believe that their behavior can effectively alleviate or modify their pain, which might lead to the feeling of helplessness and the use of maladaptive coping strategies (like catastrophizing) that impede their well-being. Moreover, it was found that both active and passive coping strategies acted as a mediator in the relationship between internal LoC and QoL, although this mediation was only partial. It indicates that the association between a belief that health is in one’s control and well-being cannot be fully explained by the choice of coping strategies. This finding is in line with previous research that showed that internal LoC buffers emotional distress on a cognitive level separately from actual behavior and thus could also be associated with QoL in ways other than through coping.^41^

Finally, our study showed that the link between powerful-others LoC and passive coping was moderated by age. An interaction effect of internal LoC and age on active coping was not detected; therefore, H5 was confirmed only partially. The results indicate that the positive association between powerful-others LoC and passive coping is stronger for relatively older individuals (middle- and older-aged adults >47 years of age) than for younger individuals. This is consistent with the notion that older (and likely more mature) individuals are more inclined than their younger counterparts to choose coping strategies that depend on the perceived controllability of pain.^15^ Interestingly, this interaction effect involves the type of LoC that so far has produced the most contradictory results with regard to the impact of perceived control on QoL.^5^^,^^8^^,^^42^

Nevertheless, other interpretations of the obtained interaction effect are possible: The study was cross-sectional, so participants of different ages represented different cohorts. Clinical practice models have changed significantly over time. Prior to the past 2 decades, the doctor–patient relationship was between a help-seeker and a doctor, with the help-seeker meant to silently comply with the decisions of the doctor. Today, patients are more actively engaged in the treatment process and want to be in control.^43^ Therefore, it cannot be ruled out that the stronger relationship between powerful-others LoC and passive coping strategies that was detected among middle- and older-aged participants could reflect the effect of being born and raised at different times and thus having different norms. Regardless of whether this effect is due to intra-individual development or to the cohort effect, our study shows that participants’ age cannot be ignored in the investigation of interrelationships among LoC, coping, and QoL.

Some limitations deserve consideration in the interpretation of our results. First, the study did not involve a formal medical diagnosis: We relied on participants’ self-reports about their chronic pain disorders. Because younger and more educated individuals prevail among Internet users, and more than a half of the studied sample were 36 years of age or younger, with only 4.6% of participants 60 years of age or older, the findings might not generalize to other populations. This is especially important, given the evidence that coping and perceived control change in later life.^44^^,^^45^ On average, participants reported moderate pain (5.72 on a 0-to-10 numeric rating scale), and data about the exact pain duration were not collected. Further studies are needed to check whether the present findings will replicate in a sample consisting of patients with more intense or long-lasting pain. Also, similar to some previous investigations of patients with chronic pain,^22^^,^^46^ the study was cross-sectional, so we cannot infer about the direction of causation. Although studies that controlled for reciprocal effects indicated that coping and individual beliefs are predictors of well-being, rather than vice versa,^41^ experimental and longitudinal studies should be carried out in the future to better understand the interplay among LoC, coping, QoL, and age. In particular, it could be valuable to review patients’ charts and history on a longitudinal basis to check whether and how their LoC, coping strategies, pain intensity, and QoL change over the course of treatment. Moreover, we did not collect racial and ethnic information in our study. We might assume that most participants were white and Polish because ethnically and racially Poland is a relatively homogenous country.^47^ Further studies should explore how the model proposed by us performs in more culturally diverse samples. Furthermore, it would be interesting to investigate the role played by religiosity in the interplay among LoC, age, coping, and QoL, as the previous research suggests this construct could possibly influence the relationship between LoC and coping,^38^^,^^39^ and it has been proved to be associated with age and QoL.^48^

## Conclusions

In conclusion, this study proposes a useful, integrative model that explains the nature of numerous associations that have been found in previous research. It also helps us to understand the mechanism through which pain LoC is linked with QoL among people suffering from chronic pain conditions. Our results (1) provide additional evidence for the importance of LoC and coping for the QoL of patients with chronic pain, (2) help us to understand interconnections among these variables, and (3) provide novel information about the moderating role of age in the LoC–coping–QoL relationship.

From a practical standpoint, the findings suggest that pain management interventions aimed at improving the well-being of patients with chronic pain should consider their control perceptions, strategies they use to cope with pain, and finally their age. The results support the notion that strengthening individuals’ belief in their own ability to manage pain and promoting active cognitive and behavioral strategies of coping with pain can be beneficial for patients’ well-being. They also add to the evidence that treatment of chronic pain should be multidimensional and should include not only pharmacological methods but also interventions aimed at shaping patients’ beliefs.^49^^,^^50^ Moreover, the findings very tentatively suggest that middle- and older-aged adults, who are used to a more paternalistic treatment model, could potentially benefit from programs encouraging a more active patient role in the medical provider–patient relationship.

## Supplementary Material

pnad079_Supplementary_Data

## Data Availability

Data can be requested for academic use by contacting the first author.
