# Supplementary material for: Age as a moderator in the interplay among locus of control, coping, and quality of life of people with chronic pain
Source: Pain Med. 2023 Jun 16;24(11):1251–61. doi: 10.1093/pm/pnad079 (PMC10628979; doi:10.1093/pm/pnad079)
Supplement: pnad079_Supplementary_Data [file pnad079_supplementary_data.zip › suppl_data/Supplementary figure PM.docx]

**Supplementary figure. Age distribution of the sample**
